# Machine learning-based prediction of candidate gene biomarkers correlated with immune infiltration in patients with idiopathic pulmonary fibrosis

**DOI:** 10.3389/fmed.2023.1001813

**Published:** 2023-02-13

**Authors:** Yufeng Zhang, Cong Wang, Qingqing Xia, Weilong Jiang, Huizhe Zhang, Ehsan Amiri-Ardekani, Haibing Hua, Yi Cheng

**Affiliations:** ^1^Department of Pulmonary and Critical Care Medicine, Jiangyin Hospital of Traditional Chinese Medicine, Jiangyin Hospital Affiliated to Nanjing University of Chinese Medicine, Jiangyin, Jiangsu, China; ^2^Department of Respiratory Medicine, Yancheng Hospital of Traditional Chinese Medicine, Yancheng Hospital Affiliated to Nanjing University of Chinese Medicine, Yancheng, Jiangsu, China; ^3^Department of Phytopharmaceuticals (Traditional Pharmacy), Faculty of Pharmacy, Shiraz University of Medical Sciences, Shiraz, Iran; ^4^Department of Gastroenterology, Jiangyin Hospital of Traditional Chinese Medicine, Jiangyin Hospital Affiliated to Nanjing University of Chinese Medicine, Jiangyin, Jiangsu, China; ^5^Department of Respiratory Medicine, Xinhua Hospital, Shanghai Jiao Tong University School of Medicine, Shanghai, China

**Keywords:** gene biomarker, immune infiltration, idiopathic pulmonary fibrosis, machine learning algorithm, CIBERSORT

## Abstract

**Objective:**

This study aimed to identify candidate gene biomarkers associated with immune infiltration in idiopathic pulmonary fibrosis (IPF) based on machine learning algorithms.

**Methods:**

Microarray datasets of IPF were extracted from the Gene Expression Omnibus (GEO) database to screen for differentially expressed genes (DEGs). The DEGs were subjected to enrichment analysis, and two machine learning algorithms were used to identify candidate genes associated with IPF. These genes were verified in a validation cohort from the GEO database. Receiver operating characteristic (ROC) curves were plotted to assess the predictive value of the IPF-associated genes. The cell-type identification by estimating relative subsets of RNA transcripts (CIBERSORT) algorithm was used to evaluate the proportion of immune cells in IPF and normal tissues. Additionally, the correlation between the expression of IPF-associated genes and the infiltration levels of immune cells was examined.

**Results:**

A total of 302 upregulated and 192 downregulated genes were identified. Functional annotation, pathway enrichment, Disease Ontology and gene set enrichment analyses revealed that the DEGs were related to the extracellular matrix and immune responses. COL3A1, CDH3, CEBPD, and GPIHBP1 were identified as candidate biomarkers using machine learning algorithms, and their predictive value was verified in a validation cohort. Additionally, ROC analysis revealed that the four genes had high predictive accuracy. The infiltration levels of plasma cells, M0 macrophages and resting dendritic cells were higher and those of resting natural killer (NK) cells, M1 macrophages and eosinophils were lower in the lung tissues of patients with IPF than in those of healthy individuals. The expression of the abovementioned genes was correlated with the infiltration levels of plasma cells, M0 macrophages and eosinophils.

**Conclusion:**

COL3A1, CDH3, CEBPD, and GPIHBP1 are candidate biomarkers of IPF. Plasma cells, M0 macrophages and eosinophils may be involved in the development of IPF and may serve as immunotherapeutic targets in IPF.

## 1. Introduction

Idiopathic pulmonary fibrosis (IPF) is not only a chronic disorder but also a progressive interstitial lung disease. The aetiology of IPF remains unclear, with its pathological presentation being usual interstitial pneumonia (UIP) (1). IPF is an infrequently diagnosed disease with an incidence of approximately 2.8–9.3 per 100,000 population in Europe and North America. Epidemiological data on IPF are scarce in China; however, its incidence has remarkably increased in recent years (2). IPF progresses gradually at the early stage, leading to diffuse fibrosis of the lungs and eventually respiratory failure and death (3). At present, a few drugs are available for treating IPF; among which, pirfenidone and nintedanib have demonstrated evident curative effects. Traditional Chinese medicine (TCM) may play a central role in managing IPF (4). Owing to the limited understanding of the pathogenesis of IPF and the lack of early intervention strategies, IPF has become a serious life-threatening disease (5). The prognosis of individuals with IPF is poor, with an estimated median survival of approximately 3 years (6). Therefore, identifying new biomarkers for the diagnosis of IPF is important for improving its treatment and prognosis.

Early and definite diagnosis of IPF is the initial step to improving the clinical treatments and survival rate of patients with IPF. To date, several biochemical markers have been associated with the occurrence of IPF and used as references for its clinical diagnosis (7, 8). However, they are inefficient for early detection of IPF owing to their limited sensitivity and specificity. Genetic factors may play a key role in the pathogenesis of IPF. IPF is a complicated and multifactorial illness that develops through the synergy of genetic and environmental factors (9, 10).

The principal processes associated with the development of IPF as a chronic lung disorder include inflammation and fibrosis. Inflammatory cytokines produced by immune cells can result in fibroblast activation, angiogenesis and connective tissue cell proliferation (11). Additionally, immune dysregulation can enhance the progression of IPF and involves numerous biomarkers associated with the prognosis of IPF (12). Studies on animals and humans have demonstrated that innate and adaptive immune processes may exacerbate the existing fibrotic responses (13).

In recent studies, microarray technology has been used in combination with machine learning algorithms to discover new genes associated with different conditions, which may serve as diagnostic and prognostic biomarkers. Additionally, scholars have suggested that immune cell infiltration, which is closely related to these disease-associated genes, plays a substantial role (14, 15). However, to date, only a few studies have employed microarray technology and machine learning algorithms to verify the role of immune cell infiltration in IPF and identify probable diagnostic markers for IPF.

In this study, three microarray datasets of IPF were extracted from the Gene Expression Omnibus (GEO) database and combined into a metadata cohort. Differentially expressed genes (DEGs) between tissues of patients with IPF and healthy individuals were identified using data from the metadata cohort. The DEGs were analysed through Gene Ontology (GO) functional annotation analysis, Kyoto Encyclopedia of Genes and Genomes (KEGG) pathway enrichment analysis, Disease Ontology (DO) enrichment analyses and gene set enrichment analysis (GSEA). Subsequently, machine learning algorithms were used for identifying candidate gene biomarkers of IPF. The identified genes were verified in a validation cohort from the GEO database. Receiver operating characteristic (ROC) curves were plotted to assess the prognostic value of the detected biomarkers in both metadata and validation cohorts. The cell-type identification by estimating relative subsets of RNA transcripts (CIBERSORT) algorithm was used to evaluate the proportion of immune cells in the lung tissues of patients with IPF and healthy individuals based on their gene expression data. Additionally, the correlation between the detected biomarkers and infiltrating immune cells was examined.

## 2. Materials and methods

### 2.1. Microarray data

The matrix files of the GSE21369, GSE24206 and GSE110147 datasets were acquired from the NCBI GEO database^1^. Data in the GSE21369 and GSE24206 datasets were acquired based on the GPL570 platform of Affymetrix Human Genome U133 Plus 2.0 Array (16, 17), whereas data in the GSE110147 dataset were acquired based on the GPL6244 platform of Affymetrix Human Gene 1.0 ST Array (18). The GSE21369 dataset included 11 lung tissue samples from patients with IPF and 6 lung tissue samples from healthy individuals. The GSE24206 dataset included 17 lung tissue samples from patients with IPF and 6 lung tissue samples from healthy donors. The GSE110147 dataset included 22 lung tissue samples from the recipient organs of patients with IPF and 11 normal lung tissue samples from tissue flanking lung cancer resections.

Probes in all datasets were transformed to gene symbols using their probe annotation files. The probe average was determined as the final expression value of genes if more than one probe corresponded to the same gene symbol. The three datasets were combined to obtain a metadata cohort for subsequent integrative analysis.

In addition, the GSE53845 dataset based on the GPL6480 platform of the Agilent-014850 Whole Human Genome Microarray 4x44K G4112F was used as the validation cohort. It included lung tissue samples from 40 patients with IPF and 8 healthy individuals (19).

### 2.2. Processing of data and screening of DEGs

The ‘SVA’ package in R was used to pre-process data in the metadata cohort and eliminate batch effects (20). The ‘limma’ package in R was used for data normalisation, background correction and identification of DEGs between 50 patients with IPF and 23 healthy individuals in the metadata cohort (21). Adjusted (adj) *p*-values of <0.05 and |log2 fold change (FC)| values of >1 were considered the threshold values for identifying significant DEGs. The ‘pheatmap’ package was used to construct a heatmap for demonstrating the expression levels of the identified DEGs.

### 2.3. Enrichment analyses of DEGs

The ‘clusterProfiler,’ ‘DOSE’ and ‘GSEABase’ packages were used for GO functional annotation, KEGG pathway enrichment and DO enrichment analyses and GSEA to examine substantial functions of the DEGs (22–25).

GO analysis incorporates three aspects, namely, molecular functions (MFs), cellular components (CCs) and biological processes (BPs). The ‘c2.cp.kegg.v7.0.symbols.gmt’ gene set from the Molecular Signatures Database (MSigDB)^2^ was used as a reference for GSEA (26, 27). The primary finding of GSEA is the enrichment score (ES), which indicates the extent to which a gene set is overexpressed at either the top or bottom of a list of ranked genes. Positive and negative ESs demonstrate gene set enrichment at the top and bottom of the ranked list, respectively. In this study, genes with |normalised ESs (NESs)| of >1, *p*-values of <0.05 and adj *p*-values of <0.25 were considered remarkedly enriched.

### 2.4. Screening of candidate gene biomarkers

To identify remarkable predictive variables, two machine learning algorithms were used to screen for genes associated with IPF. Least absolute shrinkage and selection operator (LASSO) is an algorithm of regression analysis that uses regularisation to enhance the reliability of predictions (28). LASSO analysis was performed using the ‘glmnet’ package in R to identify genes associated with the diagnosis of IPF (29). Support vector machine (SVM) is a supervised and extensively used machine-learning approach that functions in not only classification but also regression (30). To alleviate overfitting, the recursive feature elimination (RFE) algorithm was used to select optimal genes from the metadata cohort (31). To identify genes with the highest discriminative power, SVM–RFE was implemented using the ‘e1071’ and ‘kernlab’ packages in R (32, 33).

The overlapping genes between the two algorithms were defined as candidate gene biomarkers. Thereafter, the expression of these genes was verified in the GSE53845 dataset.

### 2.5. Diagnostic value of the identified gene biomarkers in IPF

To investigate the predictive value of the identified gene biomarkers, ROC curves were plotted based on the mRNA expression data of 50 patients with IPF and 23 healthy individuals in the metadata cohort. The area under the ROC curve (AUC) was evaluated to determine the diagnostic value of the genes. The AUC value was subsequently verified in the GSE53845 dataset.

### 2.6. Determination of immune cell subtypes

The CIBERSORT algorithm^3^, a bioinformatic analytical tool, was used to evaluate the relative proportion of infiltrating immune cells based on the gene expression data of patients with IPF and healthy individuals. The CIBERSORTx tool from the Alizadeh Lab and Newman Lab is used to impute gene expression profiles and estimate the abundance of member cell types in a mixed cell population using the gene expression data (34, 35). In this study, the CIBERSORTx tool was used to evaluate the abundance of 22 types of immune cells (reference set that had 1,000 permutations in the LM22 Signature Matrix file downloaded from CIBERSORTx).

Thereafter, the ‘corrplot’ in R was used to assess the distribution of the abundance of the 22 types of infiltrating immune cells and the correlation among them. The ‘vioplot’ package in R was used to construct violin plots for demonstrating differences in immune cell infiltration between patients with IPF and healthy individuals.

### 2.7. Analysis of the correlation between infiltrating immune cells and candidate genes

The correlation between the expression of candidate genes and the infiltration levels of immune cells was investigated through Spearman’s rank correlation analysis in the R program. The ‘ggplot2’ package was used to visualise the resulting relationships (36).

### 2.8. Statistical analysis

The R software (version: 4.0.3) was used for all statistical analyses. Continuous variables were compared between groups using two tests: The Student’s t-test was used to compare normally distributed variables, whereas the Mann–Whitney U test was used to compare abnormally distributed variables. The ‘glmnet’ package was used for LASSO regression analysis, whereas the ‘e1071’ and ‘kernlab’ packages in R were used for SVM–RFE. ROC curves were plotted and AUC values were evaluated to assess the diagnostic efficacy of the candidate gene biomarkers. Spearman’s correlation analysis was performed to examine the correlation between the expression of candidate genes and the infiltration levels of immune cells. All statistical tests were two-sided, and *p*-values of <0.05 were considered significant. For screening DEGs between patients with IPF and healthy individuals, adj *p*-values of <0.05 and |log2 FC| values of >1 were defined as the threshold values. For GO, KEGG and DO enrichment analyses, adj *p*-values of <0.05 were considered significant. For GSEA, genes with |NESs| of >1, *p*-values of <0.05 and adj *p*-values of <0.25 were considered significantly enriched.

## 3. Results

### 3.1. Detection of DEGs

**Figure 1:**
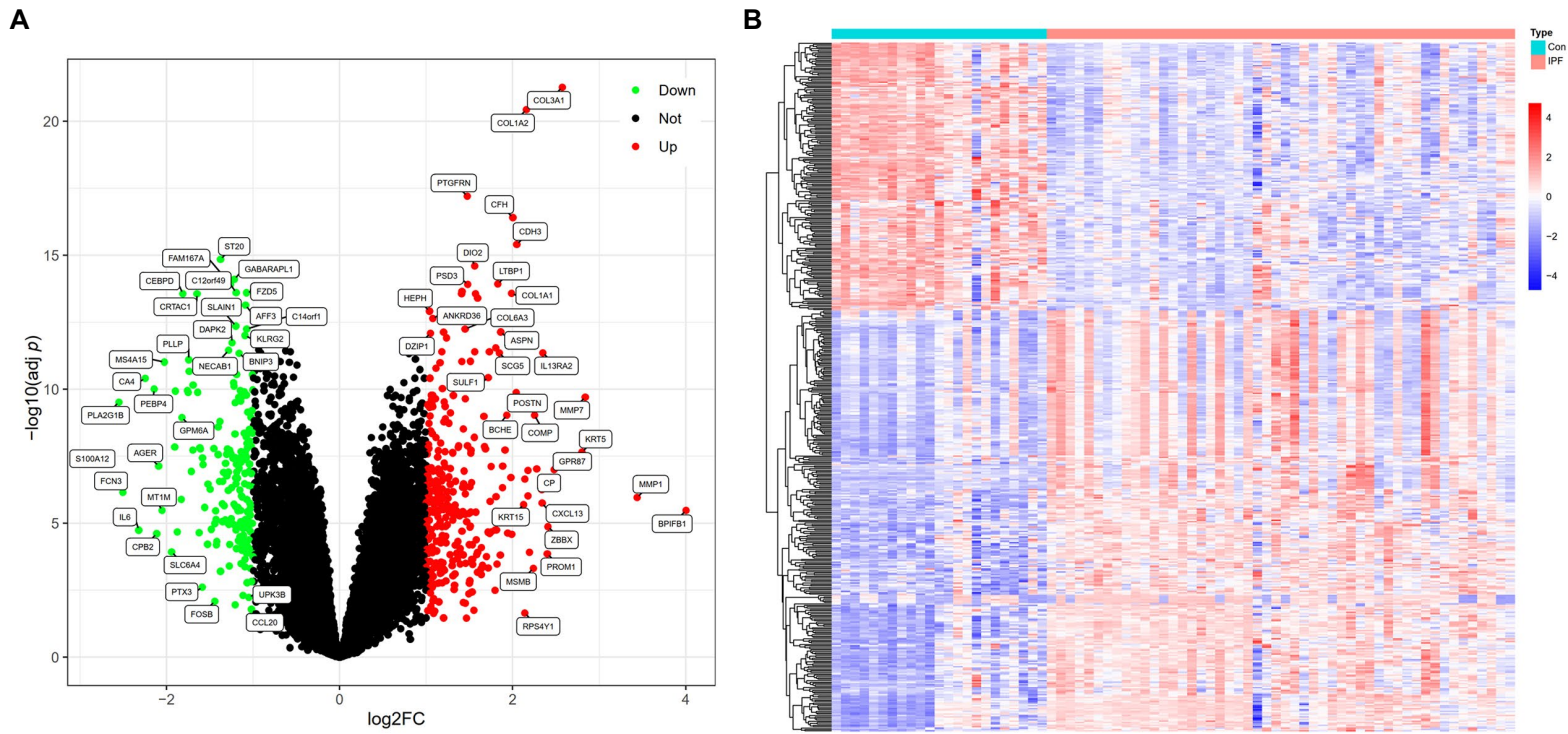
DEGs between patients with IPF and healthy individuals. **(A)** Volcano plot of DEGs identified based on the threshold of |log2FC| values of >1 and adj *p*-values of <0.05; the green (Down) and red (Up) dots represent downregulated and upregulated genes in patients with IPF, respectively; the black dots (Not) represent genes that are not differentially expressed between patients with IPF and healthy individuals. **(B)** Heatmap demonstrating the expression levels of the DEGs in 23 healthy individuals (Con) and 50 patients with IPF (IPF); red represents high expression, and blue represents low expression.

The gene expression data of 50 patients with IPF and 23 healthy individuals in the metadata cohort (GSE21369, GSE24206 and GSE110147) were retrospectively analysed (Supplementary File 1). After eliminating batch effects, DEGs between patients with IPF and healthy individuals were identified using the ‘limma’ package. Based on the threshold of adj *p*-values of <0.05 and |log2FC| values of >1, 494 DEGs were identified, including 302 upregulated (log2FC > 1) and 192 downregulated (log2FC < −1) genes (Supplementary File 2). A volcano plot and heatmap demonstrating the expression of these DEGs are shown in Figures 1A,B, respectively.

### 3.2. Enrichment analyses

**Figure 2:**
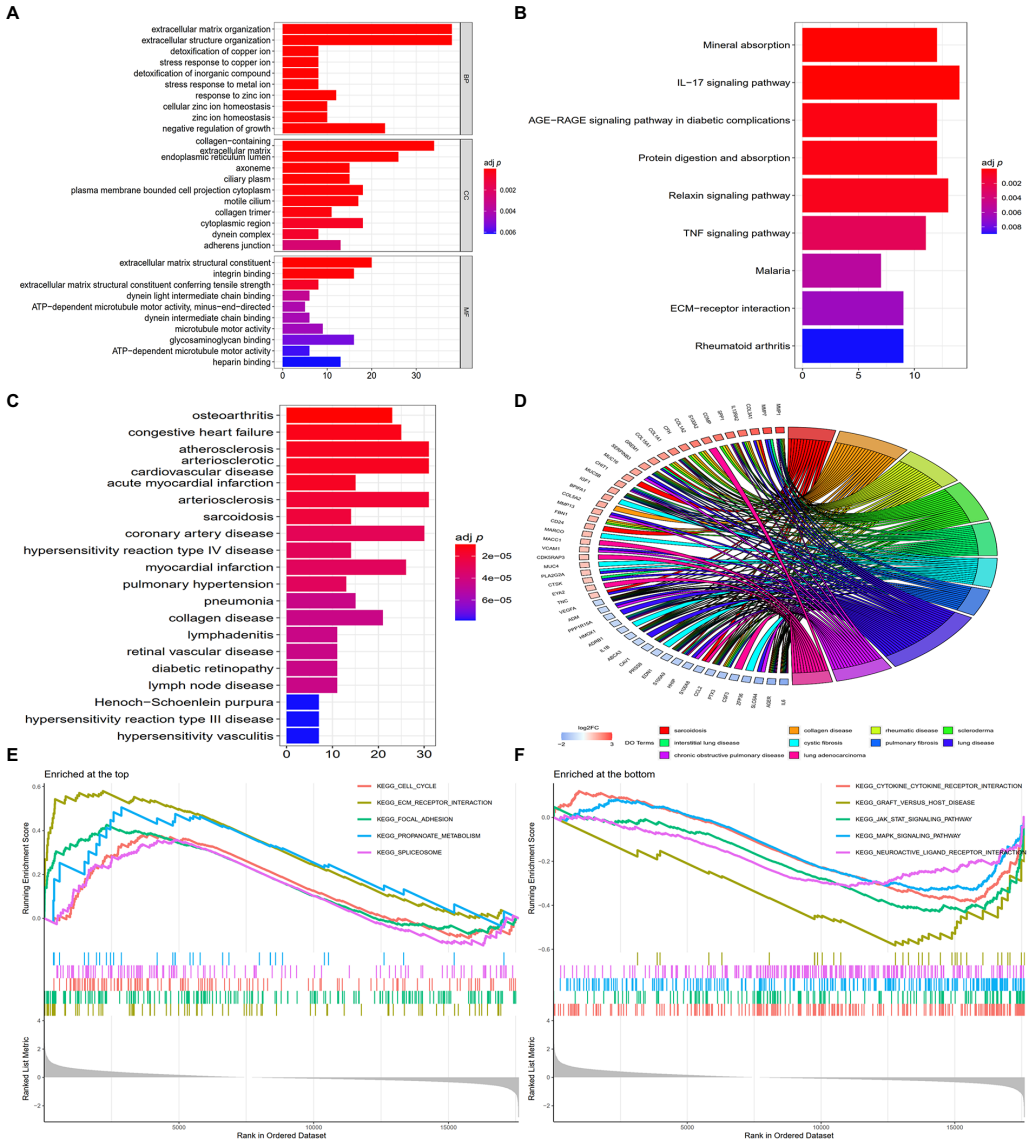
Enrichment analyses of DEGs. **(A)** The top 10 GO terms ranked based on their adj *p*-values. BP, biological process; CC, cellular component; MF, molecular function. **(B)** The nine enriched KEGG pathways. **(C)** The top 20 DO terms ranked based on their adj *p*-values. **(D)** Chord plot demonstrating the 10 main enrichments associated with IPF based on DO analysis, and gene names with the connection represent their enriched genes. **(E)** The 5 enriched gene sets at the top of the ranked list (NES > 1) indicate higher expression in IPF. **(F)** The 5 enriched gene sets at the bottom of the ranked list (NES < −1) indicate lower expression in IPF.

GO analysis revealed that the DEGs were remarkably enriched in BPs such as extracellular matrix (ECM) organisation, extracellular structure organisation, detoxification of copper ions, stress response to copper ions, detoxification of inorganic compounds and other related processes. Additionally, the DEGs were substantially enriched in CCs such as collagen-containing ECM, endoplasmic reticulum lumen, ciliary plasm, axoneme and plasmalemma-bound cell projection cytoplasm and MFs such as ECM structural constituents, integrin binding, ECM structural constituent contributing to tensile strength, dynein light intermediate chain binding and adenosine triphosphate (ATP)-dependent/minus-end-directed microtubule motor activity (Supplementary File 3). The top 10 GO terms ranked based on their adj *p*-values are shown in Figure 2A.

KEGG pathway enrichment analysis revealed that the DEGs were remarkedly enriched in pathways associated with mineral absorption, interleukin 17 (IL-17) signalling, advanced glycation end product (AGE) receptor (RAGE) signalling in diabetic complications, protein digestion and absorption, relaxin signalling, TNF signalling, malaria, ECM–receptor interaction and rheumatoid arthritis (Supplementary File 4). The top nine KEGG pathways ranked based on their adj *p*-values are shown in Figure 2B.

DO enrichment analysis was also performed to determine the functions of the DEGs. The results revealed that the DEGs were primarily associated with various illnesses (Supplementary File 5); among which, sarcoidosis, collagen disease, rheumatic disease, interstitial lung disease and pulmonary fibrosis are associated with IPF. The 20 DO terms ranked based on their adj *p*-values are shown in Figure 2C, and the 10 main diseases associated with IPF are shown in chord plots with the related genes in Figure 2D.

GSEA revealed that the DEGs were enriched in pathways associated with cytokine–cytokine receptor interaction, ECM–receptor interaction, Janus-activated kinase signal transducers, activators of transcription (JAK–STAT) signalling, mitogen-activated protein kinase (MAKP) signalling and focal adhesion (Supplementary File 6). The 5 gene sets enriched at the top of the ranked list (NES > 1) ranked based on their *p*-values are shown in Figure 2E, whereas the 5 gene sets enriched at the bottom of the ranked list (NES < −1) ranked based on their *p*-values are shown in Figure 2F.

### 3.3. Identification and validation of candidate gene biomarkers

**Figure 3:**
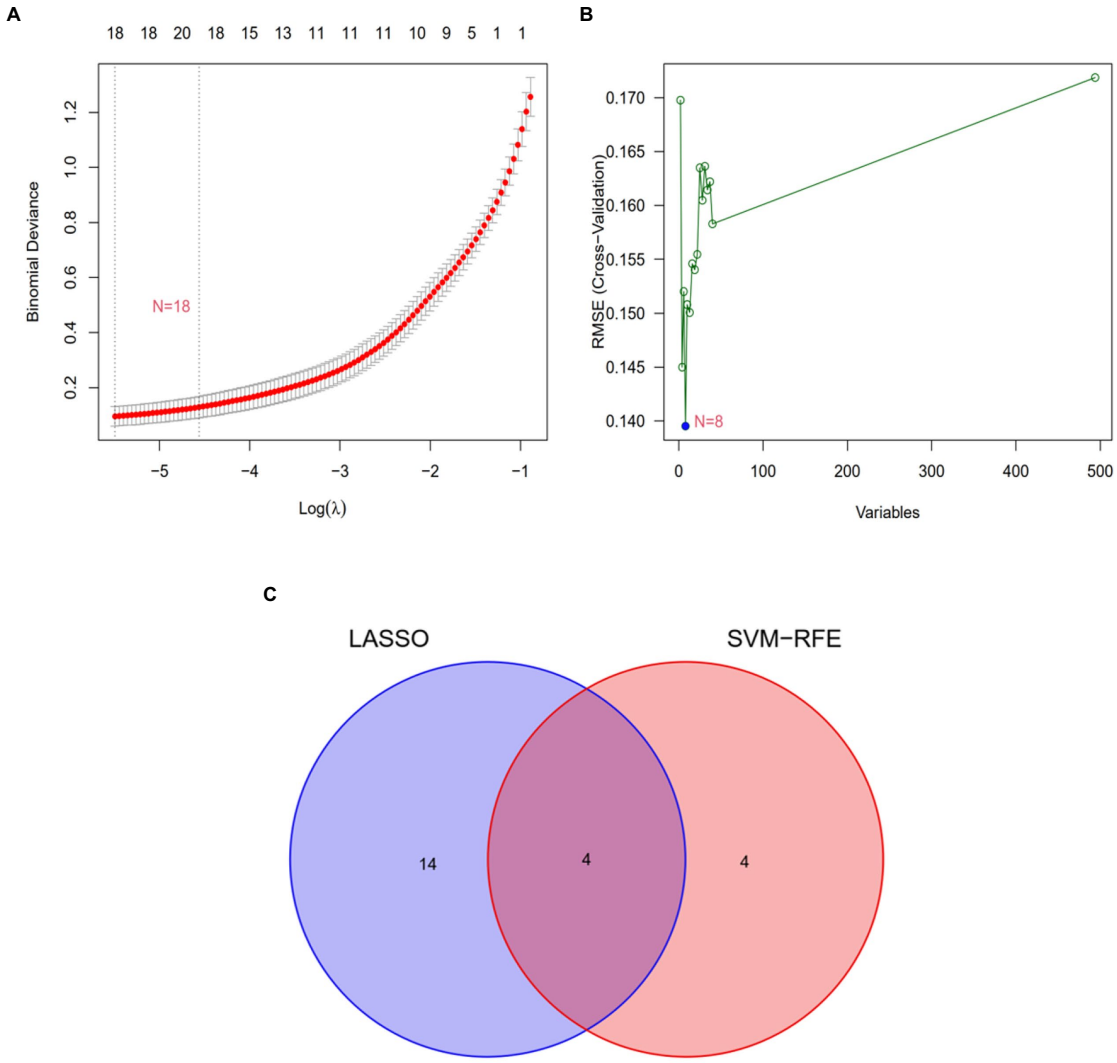
Screening of candidate gene biomarkers using two machine learning algorithms. **(A)** Tuning feature selection using the LASSO algorithm. **(B)** A plot of biomarker selection using the SVM-RFE algorithm. **(C)** Venn diagram demonstrating the four diagnostic markers (COL3A1, CDH3, CEBPD, and GPIHBP1) shared by the LASSO and SVM-RFE algorithms.

Two algorithms were used to screen for potential diagnostic biomarkers for IPF. The DEGs were screened using the LASSO regression algorithm, resulting in the identification of 18 variables as diagnostic biomarkers (Table 1; Figure 3A). A subset of eight genes among the DEGs was determined using the SVM–RFE algorithm (Table 2; Figure 3B). The four overlapping genes between these two algorithms were eventually identified as candidate diagnostic biomarkers, including collagen type III alpha 1 chain (COL3A1), cadherin 3 (CDH3), CCAAT enhancer-binding protein delta (CEBPD) and glycosylphosphatidylinositol-anchored high-density lipoprotein-binding protein 1 (GPIHBP1) (Figure 3C).

**Table 1:** Identification of 18 variables using the LASSO regression algorithm.

Gene symbol	Description
COL3A1	Collagen type III alpha 1 chain
CDH3	Cadherin 3
ST20	Suppressor of tumorigenicity 20
CEBPD	CCAAT enhancer-binding protein delta
CRTAC1	Cartilage acidic protein 1
HEPH	Hephaestin
DZIP1	DAZ-interacting zinc finger protein 1
MS4A15	Membrane spanning 4-domains a15
LOC100131541	Not applicable
GPIHBP1	Glycosylphosphatidylinositol-anchored high-density lipoprotein-binding protein 1
IRS2	Insulin receptor substrate 2
SCARNA17	Small Cajal body-specific RNA 17
LRRN1	Leucine-rich repeat neuronal 1
MYOCD	Myocardin
FNDC1	Fibronectin type III domain containing 1
CHI3L2	Chitinase 3-like 2
LYVE1	Lymphatic vessel endothelial hyaluronan receptor 1
TSPAN11	Tetraspanin 11

**Table 2:** Identification of eight variables using the SVM–RFE algorithm.

Gene symbol	Description
COL3A1	Collagen type III alpha 1 chain
TSHZ2	Teashirt zinc finger homeobox 2
COL1A2	Collagen type I alpha 2 chain
CDH3	Cadherin 3
PSD3	Pleckstrin and Sec7 domain-containing 3
CEBPD	CCAAT enhancer-binding protein delta
PTGFRN	Prostaglandin F2 receptor inhibitor
GPIHBP1	Glycosylphosphatidylinositol-anchored high-density lipoprotein-binding protein 1

**Figure 4:**
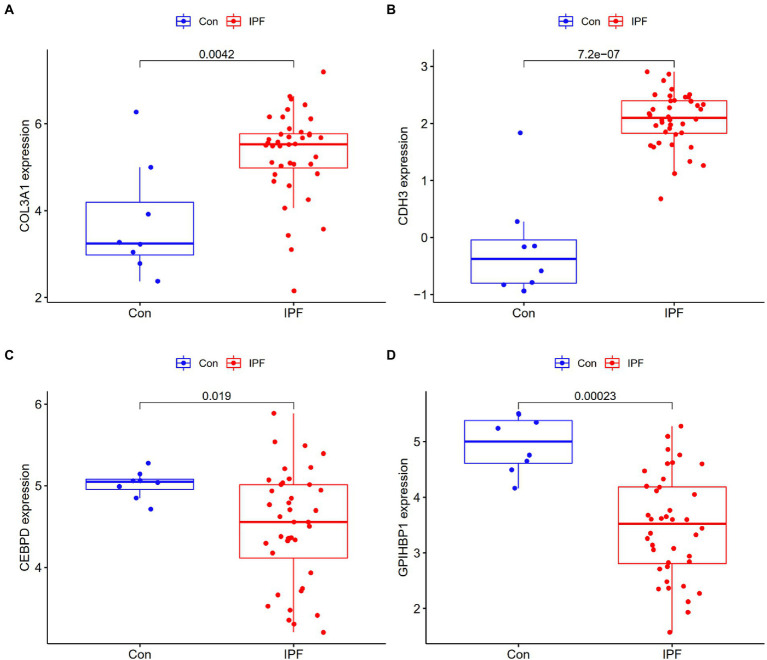
Validation of the expression of candidate genes in the GSE53845 dataset. **(A)** The expression of COL3A1 was higher in the lung tissues of patients with IPF (IPF) than in those of healthy individuals (Con). **(B)** The expression of CDH3 was higher in the lung tissues of patients with IPF than in those of healthy individuals. **(C)** The expression of CEBPD was lower in the lung tissues of patients with IPF than in those of healthy individuals. **(D)** The expression of GPIHBP1 was lower in the lung tissues of patients with IPF than in those of healthy individuals.

To assess the reliability and accuracy of the four candidate genes, their expression was verified in the GSE53845 dataset (Supplementary File 7). The expression of COL3A1 and CDH3 was higher in the lung tissues of patients with IPF than in those of healthy individuals (*p* < 0.05; Figures 4A,B), whereas the expression of CEBPD and GPIHBP1 was remarkably lower in the lung tissues of patients with IPF than in those of healthy individuals (*p* < 0.05) (Figures 4C,D). These results were consistent with those of differential expression analysis in the metadata cohort. Therefore, the four genes were considered candidate diagnostic biomarkers for further analysis.

### 3.4. Diagnostic efficiency of the four candidate biomarkers in IPF

**Figure 5:**
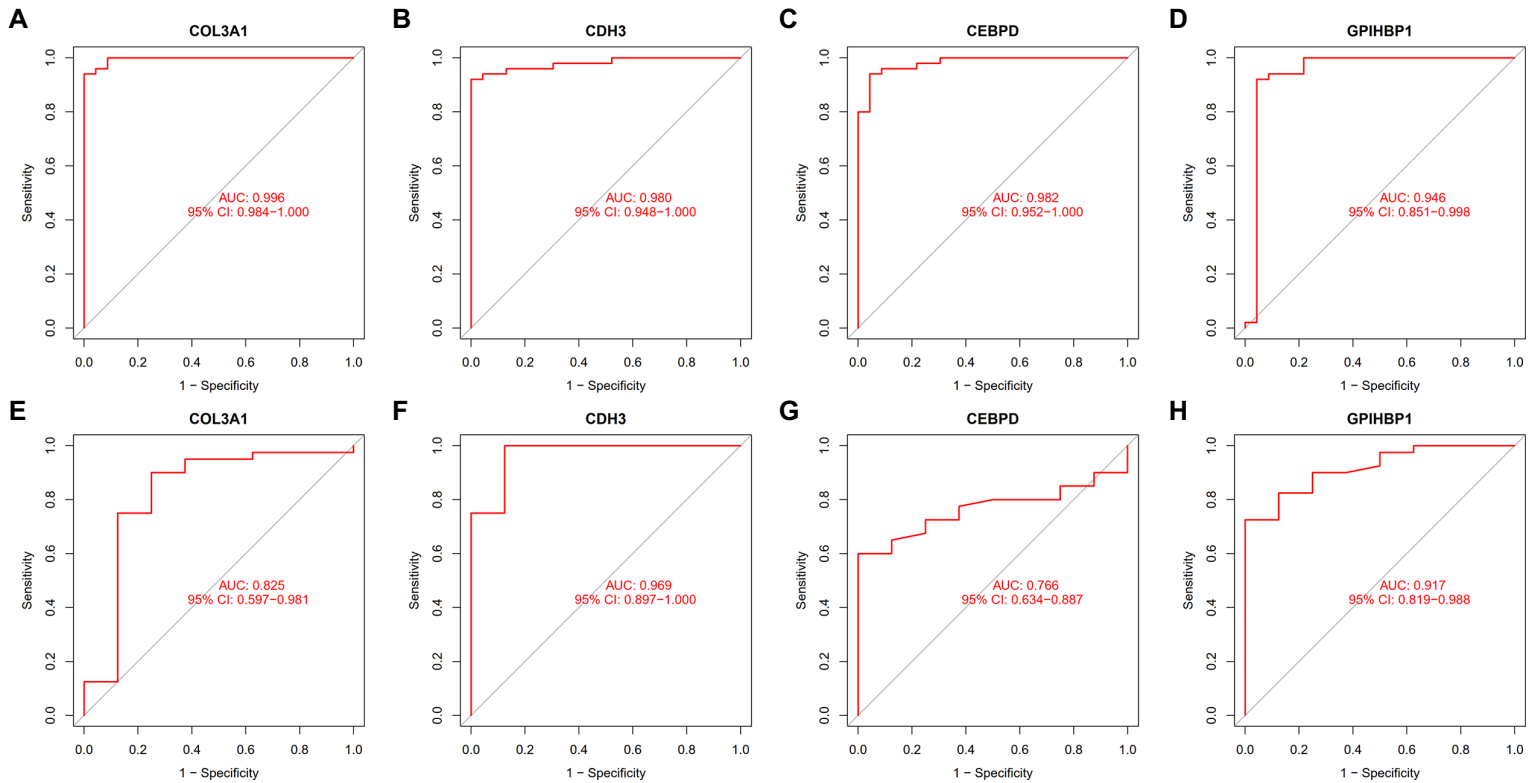
ROC curves demonstrating the diagnostic efficiency of the four candidate biomarkers. **(A)** ROC curve of COL3A1 after fitting to one variable in the metadata cohort. **(B)** ROC curve of CDH3 after fitting to one variable in the metadata cohort. **(C)** ROC curve of CEBPD after fitting to one variable in the metadata cohort. **(D)** ROC curve of GPIHBP1 after fitting to one variable in the metadata cohort. **(E)** ROC curve of COL3A1 after fitting to one variable in the GSE53845 dataset. **(F)** ROC curve of CDH3 after fitting to one variable in the GSE53845 dataset. **(G)** ROC curve of CEBPD after fitting to one variable in the GSE53845 dataset. **(H)** ROC curve of GPIHBP1 after fitting to one variable in the GSE53845 dataset.

ROC curves were plotted to examine the efficiency of the four biomarkers in distinguishing patients with IPF from healthy individuals. The AUC values of COL3A1, CDH3, CEBPD, and GPIHBP1 were 0.996 (95% CI, 0.984–1.000) (Figure 5A), 0.980 (95% CI, 0.948–1.000) (Figure 5B), 0.982 (95% CI, 0.952–1.000) (Figure 5C) and 0.946 (95% CI, 0.851–0.998) (Figure 5D), respectively, indicating that the four biomarkers had satisfactory diagnostic value. Additionally, the biomarkers had adequate discriminative capability in the GSE53845 dataset, with an AUC value of 0.825 (95% CI, 0.597–0.981) for COL3A1 (Figure 5E), 0.969 (95% CI, 0.897–1.000) for CDH3 (Figure 5F), 0.766 (95% CI, 0.634–0.887) for CEBPD (Figure 5G) and 0.917 (95% CI, 0.819–0.988) for GPIHBP1 (Figure 5H). These results suggest that the four candidate biomarkers have high diagnostic capability.

### 3.5. Immune cell infiltration

The CIBERSORT algorithm was used to evaluate the abundance of immune cells based on data extracted from the LM22 signature matrix file (Supplementary File 8). The results are shown in Supplementary File 9.

**Figure 6:**
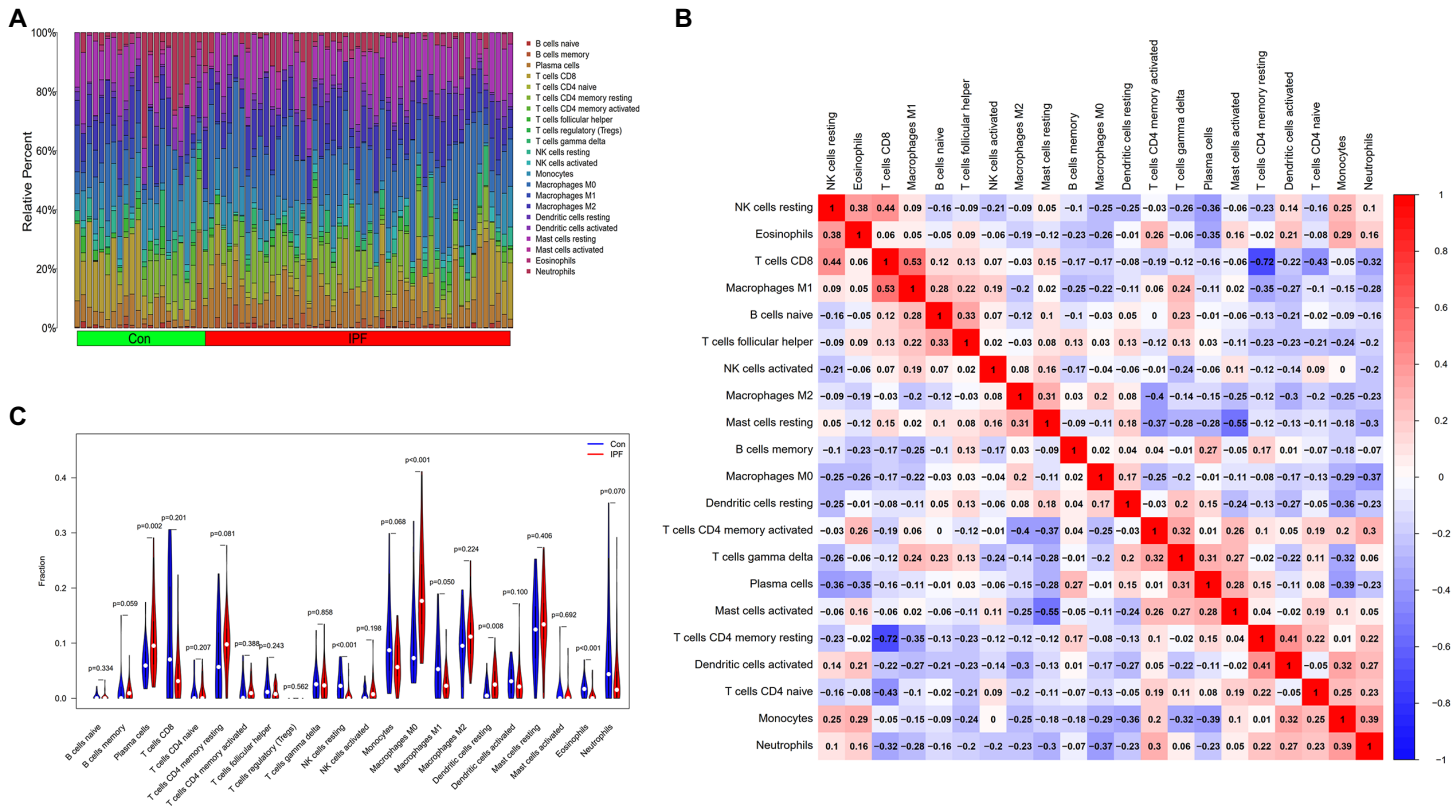
Distribution of infiltrating immune cells and the correlation among their infiltration levels. **(A)** Relative abundance of 22 immune cell subtypes in patients with IPF (IPF) and healthy individuals (Con). **(B)** Correlation among the infiltration levels of 21 immune cell subtypes (Tregs are not shown); both horizontal and vertical axes demonstrate immune cell subtypes. Red, blue and white represent higher, lower and the same correlation levels, respectively. **(C)** Comparison of the abundance of 22 immune cell subtypes between patients with IPF and healthy individuals. Blue and red colours represent the infiltration levels of healthy individuals and patients with IPF, respectively.

The distribution of 22 types of infiltrating immune cells in the IPF and control groups is demonstrated in Figure 6A. The correlation among the infiltration levels of 22 types of immune cells is demonstrated in Figure 6B (regulatory T cells [Tregs] were not correlated with any other cell and are hence not shown).

The abundance of resting natural killer (NK) cells (*p* < 0.001), M1 macrophages (*p* = 0.049) and eosinophils (*p* < 0.001) was lower in the lung tissues of patients with IPF than in those of healthy individuals. However, the abundance of plasma cells (*p* = 0.002), M0 macrophages (*p* < 0.001) and resting dendritic cells (DCs) (*p* = 0.008) was higher in the lung tissues of patients with IPF than in those of healthy individuals (Figure 6C).

### 3.6. Correlation between candidate biomarkers and infiltrating immune cells

Spearman’s rank correlation analysis was performed to examine and visualise the correlation between the expression of the four candidate genes and the infiltration levels of immune cells (Supplementary File 10).

**Figure 7:**
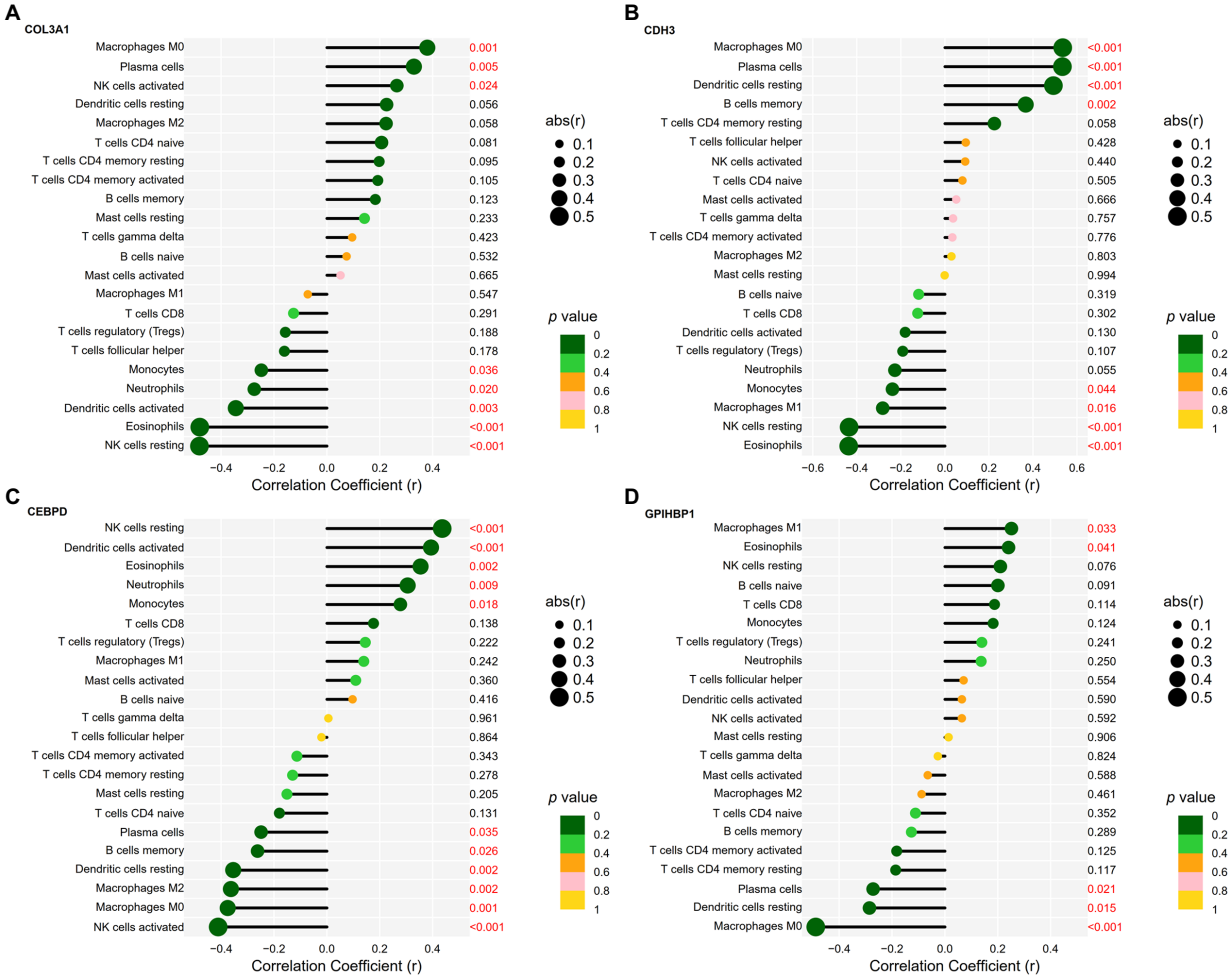
Correlation between candidate genes and infiltrating immune cells in IPF. **(A)** Correlation between COL3A1 expression and the infiltration levels of immune cells in IPF. **(B)** Correlation between CDH3 expression and the infiltration levels of immune cells in IPF. **(C)** Correlation between CEBPD expression and the infiltration levels of immune cells in IPF. **(D)** Correlation between GPIHBP1 expression and the infiltration levels of immune cells in IPF.

COL3A1 expression was positively correlated with the infiltration levels of M0 macrophages (*r* = 0.38, *p* = 0.001), plasma cells (*r* = 0.33, *p* = 0.005) and activated NK cells (*r* = 0.26, *p* = 0.024) and negatively correlated with the infiltration levels of resting NK cells (*r* = −0.48, *p* < 0.0001), eosinophils (*r* = −0.48, *p* < 0.001), activated DCs (*r* = −0.34, *p* = 0.003), neutrophils (*r* = −0.27, *p* = 0.020) and monocytes (*r* = −0.25, *p* = 0.036). The detailed results are shown in Figure 7A.

CDH3 expression was positively correlated with the infiltration levels of M0 macrophages (*r* = 0.54, *p* < 0.001), plasma cells (*r* = 0.53, *p* < 0.001), resting DCs (*r* = 0.49, *p* < 0.001) and memory B cells (*r* = 0.37, *p* = 0.002) and negatively correlated with the infiltration levels of eosinophils (*r* = −0.44, *p* < 0.001), resting NK cells (*r* = −0.44, *p* < 0.001), M1 macrophages (*r* = −0.28, *p* = 0.016) and monocytes (*r* = −0.24, *p* = 0.044). The detailed results are shown in Figure 7B.

CEBPD expression was positively correlated with the infiltration levels of resting NK cells (*r* = 0.44, *p* < 0.001), activated DCs (*r* = 0.39, *p* < 0.001), eosinophils (*r* = 0.35, *p* = 0.002), neutrophils (*r* = 0.31, *p* = 0.009) and monocytes (*r* = 0.28, *p* = 0.018) and negatively correlated with the infiltration levels of activated NK cells (*r* = −0.41, *p* < 0.001), M0 macrophages (*r* = −0.38, *p* = 0.001), M2 macrophages (*r* = −0.36, *p* = 0.002), resting DCs (*r* = −0.35, *p* = 0.002), memory B cells (*r* = −0.26, *p* = 0.026) and plasma cells (*r* = −0.25, *p* = 0.035). The detailed results are shown in Figure 7C.

GPIHBP1 expression was positively correlated with the infiltration levels of M1 macrophages (*r* = 0.25, *p* = 0.033) and eosinophils (*r* = 0.24, *p* = 0.041) and negatively correlated with the infiltration levels of M0 macrophages (*r* = −0.49, *p* < 0.001), resting DCs (*r* = −0.29, *p* = 0.015) and plasma cells (*r* = −0.27, *p* = 0.021). The detailed results are shown in Figure 7D.

## 4. Discussion

IPF is an interstitial condition characterised by UIP. At present, IPF cannot be cured and often has an unsatisfactory prognosis. Although numerous related studies have been reported, the mechanisms underlying the onset and development of IPF remain unclear (37). Epithelial–mesenchymal transition, ECM deposition and lung remodelling may be involved in the onset and progression of IPF (38–40).

Owing to the lack of biomarkers for early diagnosis of IPF, patients often miss the best opportunity for treatment, leading to progressive disease progression. Therefore, it is important to investigate the molecular mechanisms of biomarkers associated with the onset and development of IPF and identify therapeutic targets. Additionally, studies have reported that immune cell infiltration can clear ageing alveolar epithelial cells and play a role in the occurrence and development of IPF (41, 42). Therefore, the relationship between IPF-associated genes and infiltrating immune cells should be examined to improve the prognosis of IPF.

Recent studies have reported that IPF-related microRNAs (miRNAs) play an important role in the diagnosis and treatment of IPF (43–45). In previous studies, we have constructed a modulatory network of putative IPF-related miRNAs and messenger RNAs (mRNAs), which validates some miRNA–mRNA axes with TCM treatment of a bleomycin-induced IPF mouse model (4, 46). However, a few studies have examined the relationship between abnormally expressed genes and immune infiltration in IPF. In this study, we identified candidate gene biomarkers for the diagnosis of IPF and examined their correlation with immune cell infiltration in IPF.

First, three microarray datasets were extracted from the GEO database and merged into a metadata cohort, which included 50 patients with IPF and 23 healthy individuals. A total of 494 DEGs were identified, including 302 upregulated and 192 downregulated genes. GO analysis revealed the DEGs were significantly enriched in BPs such as ECM organisation, extracellular structure organisation, detoxification and stress response to copper ions and detoxification of inorganic compounds; CCs such as collagen-containing ECM, endoplasmic reticulum lumen, ciliary plasm, axoneme and plasmalemma-bound cell projection cytoplasm and MFs such as ECM structural constituent, integrin binding, ECM structural constituent conferring tensile strength, dynein light intermediate chain binding and ATP-dependent/minus-end-directed microtubule motor activity. The functions of DEGs were primarily related to ECM, indicating that the DEGs are closely related to ECM and participate in the development of IPF (38–40). KEGG analysis revealed that the DEGs were significantly enriched in pathways associated with absorption of minerals, IL-17 signalling, AGE–RAGE signalling in diabetic complications, protein digestion and absorption, relaxin signalling, TNF signalling, malaria, ECM–receptor interaction and rheumatoid arthritis. These pathways are primarily related to ECM and immune responses. DO enrichment analysis revealed that the DEGs were mainly associated with sarcoidosis, collagen disease, rheumatic disease, interstitial lung disease and pulmonary fibrosis. These diseases are associated with IPF and share some pathological characteristics with IPF. GSEA revealed that the DEGs were enriched in pathways associated with cytokine–cytokine receptor interaction, JAK–STAT signalling, ECM–receptor interaction, MAKP signalling and focal adhesion. These pathways are related to ECM, inflammation and immune responses. These findings are consistent with those of previous studies, indicating that inflammatory responses involving cytokines play a role in the pathogenesis of IPF (47–50).

With the significant advancement of science and technology, machine learning algorithms are widely used for identifying gene biomarkers and predicting disease status (51, 52). The LASSO algorithm uses regularisation to enhance the predictive accuracy (53). SVM has better performance in classification and prediction and is extensively used in disease diagnosis or medical assistance. However, it is only useful for two-group classification tasks. To avoid overfitting, the RFE algorithm can be used. Therefore, the accuracy of the classification of multiclass issues may be addressed using the SVM–RFE technique (54). CIBERSORT, a bioinformatic algorithm, is widely used to measure immune cell infiltration (34, 35). In this study, the LASSO and SVM–RFE algorithms were used to determine candidate biomarkers among the DEGs, and the CIBERSORT algorithm was used to evaluate the abundance of infiltrating immune cells in IPF.

Using the two machine learning algorithms, four candidate genes associated with the diagnosis of IPF were identified, including two upregulated genes, namely, COL3A1 and CDH3, and two downregulated genes, namely, CEBPD and GPIHBP1. The expression of these genes was verified in the validation (GSE53845) cohort. Significant differences were observed in the expression of the four genes between patients with IPF and healthy individuals in the validation cohort. These results were consistent with those of differential expression analysis in the metadata cohort. Additionally, ROC analysis revealed that the genes had a high diagnostic capability. The GSE53845 dataset contains gene expression data derived from the lung tissue samples of 40 patients with IPF and 8 healthy individuals. Because these data are derived from clinical patients, they are valid and reliable. Therefore, the abovementioned four genes were identified as candidate gene biomarkers.

COL3A1 encodes the pro-alpha 1 chains of type III collagen, which is a type of fibrillar collagen distributed in extensible connective tissues, including the skin, uterus, intestine, lung, and the vascular system, usually in association with type I collagen (55). CDH3 is a cadherin superfamily member that encodes cadherin. Multiple transcript variants are produced as a result of alternative splicing, and at least one of them encodes a preproprotein that is processed proteolytically to form a final glycoprotein. Five extracellular cadherin repeats, a greatly conserved cytoplasmic tail and a transmembrane region comprise the calcium-dependent cell–cell adhesion protein encoded by CDH3 (56). CEBPD, an intron-less gene, encodes a transcription factor with a leucine zipper domain that can attach as a homodimer to a particular DNA regulatory segment. It can also form heterodimers with CEBP-alpha, a related protein. The encoded protein plays an essential role in modulating genes involved in immune and inflammatory responses and may be involved in the modulation of genes associated with macrophage activation and/or differentiation (57). GPIHBP1 is a protein that enhances the lipolytic digestion of triglyceride-rich lipoproteins in capillary endothelial cells. It is a glycosylphosphatidylinositol-anchored lymphocyte antigen-6 family member that plays a critical role in delivering lipoprotein lipase from the subendothelial regions to the capillary lumen (58).

Dysregulated expression of COL3A1 may affect the development of IPF through regulation of IPF-related biological processes, and the expression level of COL3A1 is correlated with the prognosis of IPF (59). COL3A1 is a potential biomarker for assessing the progression of IPF and non-small cell lung cancer (NSCLC). It may help to elucidate molecular mechanisms underlying the progression of IPF and NSCLC and serve as a potential therapeutic target for IPF (60). CEBP homologous protein (CHOP) enhances alveolar epithelial cell (AEC) senescence through the nuclear factor-kappa B (NF-κB) pathway in pulmonary fibrosis (61). Additionally, it enhances the production of sonic hedgehog in type II AECs and stimulates the hedgehog signalling pathway in fibroblasts in pulmonary fibrosis (62). Hypoxia-inducible factor 1 alpha (HIF1A) can trigger endoplasmic reticulum stress and CHOP-mediated apoptosis in AECs, thereby playing a role in the development of IPF (63). Therefore, the four candidate genes as well as the abovementioned non-IPF-related genes warrant further intensive investigation.

CIBERSORT was used to evaluate the infiltration levels of immune cells in patients with IPF and healthy individuals. Several immune cell subtypes were found to be involved in key biological processes associated with IPF. The infiltration levels of plasma cells, M0 macrophages and resting DCs were higher and those of resting NK cells, M1 macrophages and eosinophils were lower in patients with IPF than in healthy individuals. These cells may be associated with the onset and progression of IPF.

Inflammatory and immune cells play an important role in the progression of IPF. Some results of this study are consistent with those of previous studies. The expression of FK506-binding protein (FKBP) prolyl isomerase 11 (FKBP11) is elevated in the lung tissues of patients with IPF, and FKBP11 specifically localises to antibody-producing plasma cells (64). In a study, compared with control mice, bleomycin-treated mice had an increased proportion of pulmonary IgA(+) germinal centres and plasma cells, and autoreactive IgA was identified as a diagnostic biomarker for IPF (65). M1 macrophages play a crucial role in wound healing following alveolar epithelial damage, whereas M2 macrophages are necessary for resolving inflammatory responses that develop in the lung. IPF is a pathological outcome resulting from disrupted wound healing in response to repeated injury to the lung (66). NF-κB facilitates the production of proinflammatory cytokines to exacerbate M1 macrophage polarisation (67). Pirfenidone suppresses transforming growth factor-β, which is associated with M2 macrophage polarisation and fibroblast activation and has anti-fibrotic properties (68). Polarised M1 macrophages can be converted to M0 macrophages after 12 days of incubation in a cytokine-insufficient medium or re-differentiated into a different cell phenotype after being cultured further in a different polarising medium (69). DCs are major contributors to the pathogenesis of IPF (70). In bleomycin models, lung DCs are important proinflammatory cells that maintain pulmonary inflammation and fibrosis (71). Fms-related receptor tyrosine kinase 3 ligand is overexpressed in the serum and lung tissues of patients with IPF and may facilitate the accumulation of lung DCs during pulmonary fibrogenesis (72). The proportion of resting NK cells is lower in the lung tissues of patients with IPF than in those of healthy individuals (73). Eosinophil is a principal source of several crucial pro-fibrogenic cytokines, especially in the initial stages of fibrosis (74).

COL3A1 may serve as a molecular biomarker for assessing prognosis and immune infiltration in pan-cancer (75). Collagen genes play an important role in regulating the immunosuppressive microenvironment and epithelial–mesenchymal transition in glioma and may serve as therapeutic targets for glioma (76). Biomarkers associated with collagen synthesis and degradation have the potential to enhance clinical trials in IPF and may be used for prognostic assessment and therapeutic decision-making in clinical settings (77). CDH3 is associated with immune infiltration in papillary thyroid carcinoma (78). CEBPD has been identified as a diagnostic biomarker for nonalcoholic fatty liver disease using machine learning algorithms and is associated with immune cell infiltration (79). In this study, the expression of COL3A1, CDH3, CEBPD and GPIHBP1 was correlated with the abundance of various immune cells including plasma cells, M0 macrophages and eosinophils. In particular, the expression of CDH3, CEBPD and GPIHBP1 was correlated with the abundance of resting DCs; the expression of COL3A1, CDH3 and CEBPD was correlated with the abundance of resting NK cells and the expression of CDH3 and GPIHBP1 was correlated with the abundance of M1 macrophages. The relationship of the four genes with these immune cells has been reported in some related studies. The infiltration of plasma cells has been associated with the expression of CDH3 and CEBPD (80, 81), whereas that of macrophages has been associated with the expression of COL3A1, CDH3 and CEBPD in multiple diseases (80, 82–84). In-depth experimental studies should be conducted to investigate the relationship between the four genes and immune cells in IPF.

Although this study was rigorous, its limitations should also be acknowledged. Although we collected as many samples as possible by combining the three datasets, the sample size of the metadata cohort is small. Additionally, the sample size of the validation cohort is also small. Because the role of the four biomarkers and infiltration of immune cells in IPF were examined using bioinformatic algorithms, in-depth studies with large sample size should be conducted to validate the findings. We will verify the results in a clinical cohort in future studies, with immunohistochemical detection of lung transplant specimens. Additionally, we will perform single-cell RNA sequencing on lung tissue and blood samples to verify whether the expression of the four genes is altered in immune cell clusters.

## 5. Conclusion

COL3A1, CDH3, CEBPD, and GPIHBP1 are potential biomarkers for the diagnosis of IPF. Plasma cells, M0 macrophages and eosinophils (associated with these four genes) may be involved in the development of IPF and serve as immunotherapeutic targets for the treatment of IPF.

## Data availability statement

The original contributions presented in the study are included in the article/Supplementary material, further inquiries can be directed to the corresponding authors.

## Author contributions

YZ, HZ, EA-A, HH, and YC conceived and designed the study and wrote the manuscript. YZ, CW, QX, WJ, and HZ were responsible for data collation and analysis. YZ, EA-A, HH, and YC supervised the study. YZ, HZ, and YC revised the manuscript. All authors have read and approved the final version of the manuscript.

## Funding

This work was supported by the Research Grants of Jiangyin Hospital of Traditional Chinese Medicine (202013 to WJ, 202014 to YZ), Grants from the Wuxi Health Commission’s Scientific Research Project (M202154 to YZ, T202130 to WJ), the ChengXing Talent Training Plan of Jiangyin Hospital of Traditional Chinese Medicine (2022 to YZ), Grants from the Traditional Chinese Medicine Science and Technology Development Plan Project of Jiangsu Province (ZT202113 to HH) and the National Natural Science Foundation of China (No. 82000039 to YC).

## Conflict of interest

The authors declare that the research was conducted in the absence of any commercial or financial relationships that could be construed as a potential conflict of interest.

## Publisher’s note

All claims expressed in this article are solely those of the authors and do not necessarily represent those of their affiliated organizations, or those of the publisher, the editors and the reviewers. Any product that may be evaluated in this article, or claim that may be made by its manufacturer, is not guaranteed or endorsed by the publisher.

## Footnotes

^1^ http://www.ncbi.nlm.nih.gov/geo/

^2^ http://www.gsea-msigdb.org/gsea/msigdb

^3^ https://cibersortx.stanford.edu/
